# 微滴数字PCR和Super-ARMS检测晚期肺腺癌患者血浆循环肿瘤DNA表皮生长因子受体基因突变的临床价值研究

**DOI:** 10.3779/j.issn.1009-3419.2020.02.03

**Published:** 2020-02-20

**Authors:** 喆 曹, 静 王, 娜 秦, 琨 李, 嘉林 吕, 敬慧 王, 新杰 杨, 曦 李, 卉 张, 权 张, 洪清 龙, 诚荣 舒, 丽 马, 树才 张

**Affiliations:** 1 437100 咸宁，湖北科技学院临床医学院肿瘤和核医学教研室/湖北科技学院附属第一医院肿瘤中心 Department of Cancer and Nuclear Medicine, School of Clinical Medicine, Hubei University of Science and Technology/Cancer Center, First Affiliated Hospital of Hubei University of Science and Technology, Xianning 437100, China; 2 361101 厦门，厦门市中医院医疗美容科 Department of Medical Cosmetology, Xiamen Hospital of T.C.M, Xiamen 361001, China; 3 101149 北京，首都医科大学附属北京胸科医院，北京市结核病胸部肿瘤研究所肿瘤内科 Beijing Chest Hospital Affiliated to Capital Medical University/ Department of Oncology, Beijing Institute of Tuberculosis and Thoracic Oncology, Beijing 101149, China; 4 101149 北京，首都医科大学附属北京胸科医院，北京市结核病胸部肿瘤研究所病理科 Beijing Chest Hospital Affiliated to Capital Medical University/ Department of Pathology, Beijing Institute of Tuberculosis and Thoracic Oncology, Beijing 101149, China

**Keywords:** 肺肿瘤, 微滴数字聚合酶链反应, 表皮生长因子受体, 基因突变, 循环肿瘤脱氧核糖核酸, 突变扩增系统, Super-突变扩增系统, Lung tumor, Droplet digital polymerase chain reaction, Epidermal growth factor receptor, Genetic mutations, Circulating tumor DNA, Mutation amplification system, Super-mutation amplification system

## Abstract

**背景与目的:**

晚期肺腺癌患者在选择靶向药物时需以肿瘤表皮生长因子受体（epidermal growth factor receptor, *EGFR*）基因突变类型作为依据，然而晚期肺腺癌肿瘤组织取材较难，有专家共识指出外周血可替代肿瘤组织作为检测标本。本文旨在探讨微滴数字聚合酶链反应法（droplet digital polymerase chain reaction, ddPCR）和超级扩增阻滞突变系统（super-amplification refractory mutation system, super-ARMS）这两种方法检测晚期肺腺癌患者外周血中血浆循环肿瘤脱氧核糖核酸（circulating tumor DNA, ctDNA）中*EGFR*基因突变的临床价值。

**方法:**

收集2016年2月-2019年2月首都医科大学附属北京胸科医院确诊的初治晚期肺腺癌共119例纳入研究，比较ddPCR和Super-ARMS技术检测血浆ctDNA *EGFR*基因突变的敏感度、特异度。部分*EGFR*基因经典突变阳性患者接受一线EGFR酪氨酸激酶抑制剂（EGFR tyrosine kinase inhibitors, EGFR-TKI）口服治疗，将患者按检测结果分亚组，组1为ddPCR及Super-ARMS检测*EGFR*基因突变结果均为阳性，21例。组2为ddPCR及Super-ARMS检测*EGFR*基因突变结果均为阴性，16例。组3为ddPCR检测结果为阳性而Super-ARMS检测结果为阴性，5例。组4为ddPCR检测结果为阴性而Super-ARMS检测结果阳性，因组4患者个数为0，不纳入统计。以客观缓解率（objective response rate, ORR）及疾病控制率（disease control rate, DCR）评估近期疗效，并用生存分析比较组间无进展生存时间（progression-free survival, PFS）以评估远期疗效。

**结果:**

119例晚期肺腺癌组织样本中，共检测到58例（48.7%）*EGFR*基因突变。ddPCR技术检测与肿瘤组织*EGFR*基因突变结果的符合率为82.4%（Kappa=0.647, *P* < 0.001），灵敏度为74.1%，特异度为90.2%。而Super-ARMS检测与组织检测的符合度为71.4%，灵敏度仅为58.6%，特异度为83.6%。组3的ORR与DCR值低于组1、2，但组间ORR相比均无统计学意义（*P* > 0.05）。生存分析显示，三组患者PFS比较，差异无统计学意义（*χ*^2^=2.221, *P*=0.329）。

**结论:**

ddPCR作为一种高敏感度及特异度的液体基因检测方法，可以作为检测晚期肺腺癌患者血浆ctDNA *EGFR*基因突变的一种较为可靠的方法。血浆基因检测结果同样可作为EGFR-TKIs药物对患者的疗效预测的依据。

## Abstract (en)

**Background and objective:**

The patients with advanced lung adenocarcinoma should select targeted drugs based on the type of tumor epidermal growth factor receptor (*EGFR*) gene mutation. However, it is difficult to collect tumor tissue of advanced lung adenocarcinoma, and some experts agree that peripheral blood can be used as a substitute for tumor tissue as a test specimen. This paper aimed to investigate the clinical value of ddPCR and super-amplification refractory mutation system (ARMS) in detecting *EGFR* gene mutation in peripheral blood of patients with advanced lung adenocarcinoma.

**Methods:**

A total of 119 patients diagnosed in Beijing Chest Hospital Affiliated to Capital Medical University from February 2016 to February 2019 were collected, and the sensitivity and specificity of plasma ctDNA *EGFR* gene mutation detected by ddPCR and super-arms were compared. Some patients with positive *EGFR* gene mutations received oral treatment with first-line EGFR tyrosine kinase inhibitors (EGFR-TKI). The patients were divided into subgroups according to the test results. In group 1, both ddPCR and super-arms showed positive *EGFR* gene mutation results, with 21 cases. In group 2, ddPCR and super-arms detection of *EGFR* gene mutation were all negative, with 16 cases. In group 3, the ddPCR test was positive and the super-arms test was negative, with 5 cases. In group 4, the ddPCR test result was negative while the super-arms test result was positive. Since the number of patients in group 4 was 0, no statistics were included. Objective response rate (ORR) and disease control rate (DCR) were used to evaluate the short-term outcome, and progression-free survival (PFS) was compared with survival analysis to evaluate the long-term outcome.

**Results:**

*EGFR* mutations were detected in 58 (48.7%) of 119 patients with advanced lung adenocarcinoma. The coincidence rate between ddPCR and *EGFR* gene mutation in tumor tissues was 82.4% (Kappa=0.647, *P* < 0.001), the sensitivity was 74.1%, and the specificity was 90.2%. However, the coincidence degree of super-arms test and tissue test was 71.4%, the sensitivity was only 58.6%, and the specificity was 83.6%. The ORR and DCR values in group 3 were lower than those in group 1 and 2, but there was no significant difference in ORR between groups (*P* > 0.05). Survival analysis showed that the PFS of the three groups was compared. The difference was not statistically significant (*χ*^2^=2.221, *P*=0.329).

**Conclusion:**

ddPCR, as a high sensitivity and specificity liquid gene detection method, can be used as a reliable method to detect the mutation of plasma ctDNA *EGFR* gene in patients with advanced lung adenocarcinoma. The results of plasma genetic testing can also be used as the basis for predicting the efficacy of EGFR-TKIs in patients.

表皮生长因子受体（epidermal growth factor receptor, *EGFR*）基因突变状态对晚期肺腺癌患者合理选择靶向药物至关重要。由于心肺功能差，无法取材、取材组织少或为坏死组织、病变位置特殊或质地坚韧活检钳不易着位等原因，晚期肺腺癌患者在初治或进展后组织标本基因检测困难。2018版中国非小细胞肺癌（non-small cell lung cancer, NSCLC）血液EGFR检测专家共识指出，外周血可作为检测*EGFR*基因突变的标本。现阶段临床常用Super-扩增阻滞突变系统（amplification refractory mutation system, ARMS）法来检测外周血游离肿瘤DNA的*EGFR*基因突变，其具有操作方便、费时少等优势，然而其灵敏度有待提高^[1]^。当血液标本中*EGFR*基因突变的丰度较低时，会造成一定的假阴性，使一部分患者*EGFR*基因突变被漏检。多项研究表明微滴数字PCR（droplet digital polymerase chain reaction, ddPCR）在检测循环肿瘤脱氧核糖核酸（circulating tumor DNA, ctDNA）时灵敏度可以达到0.01%，高于Super-ARMS法0.2%的灵敏度^[2-4]^。

本研究通过ddPCR法和Super-ARMS法检测119例晚期肺腺癌患者血液标本*EGFR*基因突变状况，分析比较ddPCR和Super-ARMS技术检测血浆ctDNA *EGFR*基因突变的敏感度、特异度，结合EGFR酪氨酸激酶抑制剂（tyrosine kinase inhibitors, TKI）药物疗效分析，为临床选择更佳的*EGFR*基因突变检测方法提供依据。

## 1 对象与方法

### 1.1 研究对象

收集2016年2月-2019年2月首都医科大学附属北京胸科医院确诊的初治晚期肺腺癌癌患者119例。入选标准：（1）病理确诊为肺腺癌; （2）临床分期Ⅲb期-Ⅳ期（根据第8版TNM分期）; （3）应用一代EGFR-TKI治疗前有充足的血液标本用于本研究，不干预临床实践; （4）*EGFR*敏感突变的患者接受EGFR-TKI药物口服治疗; （5）有完整的临床病理信息，包括吸烟、性别、年龄等。排除标准：（1）年龄 < 18岁; （2）怀孕女性。4例混有其他肿瘤成分，2例治疗过程中出现严重并发症死亡，被排除。纳入研究119例晚期肺腺癌患者。本研究是一项回顾性、单中心临床研究，由北京胸科医院伦理委员会批准（批号：BJC1216）。患者均知情同意并签署同意书。中国临床试验注册中心注册号ChiCTR2000029773。

### 1.2 仪器及试剂

M×3000P即时荧光定量PCR仪（Aglient公司）; QX200 Droplet微滴生成仪、读取仪（Bio-Rad公司）; 微滴化试剂（Bio-Rad公司）; Qubit^TM^荧光定量仪（Thermo Fisher Scientific公司）; Applied Biosystems 9700 PCR仪（Thermo Fisher Scientific公司）; QIAamp^®^ Circulating Nucleic Acid Kit（Qiagen公司）; ADx-Super-ARMS *EGFR*基因位点检测试剂盒（厦门艾德生物医药科技股份有限公司）; 人血液样本EGFR基因检测试剂盒（上海源奇生物医药科技有限公司）。

### 1.3 方法

#### 1.3.1 收集组织标本和血液标本

所有患者通过CT导引下经皮肺肿物穿刺或经支气管镜取活检方法收集肿瘤组织标本。收集到的标本使用4%甲醛溶液浸泡，经包埋固定后制成石蜡切片。苏木精伊红（HE）染色，在显微镜下病理学专家进行细胞类型确诊，保证至少80%以上的肿瘤细胞成分，通过显微切割移除非肿瘤成分。QIAampDNA试剂盒定量提取石蜡切片肿瘤组织的DNA。血液样本的收集：在应用靶向药物治疗前及取组织标本24 h前后利用EDTA抗凝管，抽取10 mL的静脉血液样本，离心后分离4 mL血浆。血浆游离肿瘤DNA采用QIAamp^®^ Circulating Nucleic Acid Kit提取。上述提取的DNA样本用Qubit^TM^荧光定量仪测浓度。

#### 1.3.2 ddPCR检测血浆*EGFR*基因突变

应用ddPCR技术检测血浆ctDNA *EGFR*基因突变类型。将DNA样本加入含有相应探针的ddPCR超级混合液中。充分混合并离心至20 mL ddPCR反应液体系中，其中包含有探针、模板以及引物等，样本在QX200 ddPCR系统中反应。设置反应条件：95 ℃孵育5 min，扩增30个循环，72 ℃延伸7 min后结束，降至4 ℃。通过quantasoft软件进行结果分析，阳性界值定义为 > 2个突变微滴数。

#### 1.3.3 Super-ARMS技术检测患者肿瘤组织和血浆中*EGFR*基因突变类型

通过Super-ARMS技术检测患者肿瘤组织和血浆中*EGFR*基因突变类型，厦门艾德公司ADx-ARMS试剂盒检测29种突变类型，此部分结果均从北京胸科医院病理科获得。通过Super-ARMS技术检测血浆*EGFR*基因突变类型，根据上海源奇生物医药有限公司血浆PCR应用美国Applied Biosystems公司的7500型实时定量PCR系统进行检测。试剂盒设有内部和外部质控样本，阳性对照和阴性对照*EGFR*基因突变定量检测试剂盒说明进行检测。

### 1.4 统计学方法

采用SPSS 19.0软件进行统计学分析。血浆ctDNA *EGFR*基因突变结果的敏感度与特异度以肿瘤组织检测结果为金标准。ddPCR和Super-ARMS检测*EGFR*基因状态一致性评价采用*Cohen′s Kappa*检验; 组间差异运用卡方检验。依据RECIST 1.1标准进行肿瘤治疗疗效评估。卡方检验比较ORR及DCR。通过*Kaplan-Meier*检验分析患者无进展生存期（progression-free survival, PFS）。PFS指有*EGFR*基因敏感突变的肺癌患者从接受EGFR-TKI治疗开始，到观察到疾病进展或发生因任何原因死亡的时间。末次随访时间为2019年6月18日。中位随访时间为19个月（2.4个月-40.3个月）。组间生存比较，*P* < 0.05为差异有统计学意义。

## 2 结果

### 2.1 临床病理特征

本研究共纳入患者119例，其中男63例（52.9%），女56例（47.1%）。中位年龄66岁， < 65岁者共54例（45.4%），≥65岁共65例（54.6%）。不吸烟或少吸烟者79例（66.4%），吸烟者40例（33.6%）。入组后组织检测*EGFR*基因突变的患者有58例，接受EGFR-TKIs口服治疗者42例。此42例均为EGFR基因经典突变患者，其中19-DEL突变患者22例，L858R突变患者20例。42例患者均接受一代EGFR-TKIs药物治疗，其中吉非替尼16例，厄洛替尼6例，埃克替尼20例。其中有2例患者组织突变检出阴性但血浆基因突变检出阳性，分别接受了吉非替尼和埃克替尼治疗。

### 2.2 肺腺癌组织*EGFR*基因突变检测结果

119例患者肿瘤组织标本中检测到58例（48.7%）有*EGFR*基因突变。其中，26例（44.8%）含Exon 19 del突变，其中2例合并有Exon 20 T790M突变; 27例（46.6%）含Exon 21 L858R突变; 4例（6.9%）Exon 18 G719X突变，其中2例合并Exon 21 L861Q突变。另有1例（1.7%）Exon 20 Ins插入突变。以此结果作为金标准，检验ddPCR及Super-ARMS两种方法的敏感度及特异度。

### 2.3 血浆*EGFR*基因突变检测结果

ddPCR技术检测到*EGFR*基因突变49例（41.2%），与肿瘤组织*EGFR*基因突变结果的符合率为82.4%（Kappa=0.647, *P* < 0.001），灵敏度为74.1%，特异度为90.2%。而应用Super-ARMS技术检测到血浆ctDNA中*EGFR*基因突变44例（37.0%）。以组织检测结果作为金标准，血液Super-ARMS检测灵敏度为58.6%，特异度为83.6%，与组织检测的符合度为71.4%（Kappa=0.425, *P* < 0.001）（表 1）。

**1:** 肺腺癌患者肿瘤组织及血浆检测中*EGFR*基因突变结果比较 Comparison of *EGFR* gene mutations in tumor tissues and plasma of lung adenocarcinoma patients

		Tumor tissue		Total
		+	-	
dd PCR	+	43	6	49
	-	15	55	70
SUPER-ARMS	+	34	10	44
	-	24	51	75
Total		58	61	119
Super-ARMS: super-amplification refractory mutation system; ddPCR: droplet digital polymerase chain reaction.				

血浆标本中使用ddPCR及Super-ARMS方法检测*EGFR*突变，阳性结果中同时检出19-Del突变共14例，但其中2例组织检测结果为野生型; ddPCR检出而Super-ARMS技术未检出共10例，此10例组织检测19-Del突变均为阳性; ddPCR未检出而Super-ARMS技术检出共2例，此2例组织检测结果均为阴性; 两种方法检测19-del均为阴性共44例，其中4例组织检测19-del突变阳性。以组织检测结果为金标准，ddPCR结果与组织的符合率达91.4%，Super-ARMS结果与组织符合率为74.3%（表 2）。

**2:** Super-ARMS和ddPCR检测血浆ctDNA中*EGFR*突变基因19-Del结果比较 Comparison of *EGFR* mutant gene 19-del in plasma ctDNA detected by Super-ARMS and ddPCR

19-Del		SUPER-ARMS		Total
		+	-	
ddPCR	+	14	10	24
	-	2	44	46
Total		16	54	70

血浆标本中使用两种方法阳性结果中同时检出L858R突变共16例，但其中3例组织检测结果为野生型; ddPCR检出而Super-ARMS技术未检出共6例，此6例组织检测L858R突变5例阳性，1例野生型; ddPCR未检出而ARMS技术检出共10例，此10例组织检测结果6例阳性，4例阴性; 两种方法检测L858R均为阴性共38例，其中3例组织检测L858R突变阳性。以组织检测结果为金标准，ddPCR结果与组织的符合率达81.4%，Super-ARMS结果与组织符合率为78.5%（表 3）。

**3:** Super-ARMS和ddPCR检测血浆ctDNA中*EGFR*突变基因L858R结果比较 Comparison of *EGFR* mutated gene L858R in plasma ctDNA detected by super-ARMS and ddPCR

L858R		Super-ARMS		Total
		+	-	
ddPCR	+	16	6	22
	-	10	38	48
Total		26	44	70

### 2.4 组织与血浆*EGFR*经典基因突变患者接受一代EGFR-TKI治疗后疗效分析

在所有组织中检测出*EGFR*突变阳性的患者中，有42例检出*EGFR*经典基因突变的患者（即19-DEL与L858R）接受了一代EGFR-TKIs的治疗。将这些患者进行亚组比较，组1为ddPCR及Super-ARMS检测*EGFR*基因突变结果均为阳性，21例。组2为ddPCR及Super-ARMS检测*EGFR*基因突变结果均为阴性，16例。组3为ddPCR检测结果为阳性而Super-ARMS检测结果为阴性，5例。组4为ddPCR检测结果为阴性而Super-ARMS检测结果阳性，因组4患者个数为0，不纳入统计。近期疗效以客观缓解率（objective response rate, ORR）及疾病控制率（disease control rate, DCR）为标准进行评估。以患者PFS时长作为评估远期疗效的标准进行统计。

近期疗效评价：1组的ORR为71.43%，DCR为90.48%;2组ORR为62.5%，DCR为93.75%;3组ORR为60.00%，DCR为80.00%（表 4）。组3的ORR与DCR值低于组1、2，但组间ORR相比均无统计学意义（*P* > 0.05）。远期疗效评价，接受一代EGFR-TKI治疗的患者中位PFS为12.4个月（95%CI: 10.6-16.9）; 1组中位PFS为12.4个月（95%CI: 9.9-14.9），2组中位PFS为12.7个月（95%CI: 12.2-13.2），3组中位PFS为15.8个月（95%CI: 7.9-32.5）（表 4），用*Kaplan-Meier*法分析各组PFS，结果见图 1。三组患者PFS比较差异无统计学意义（*χ*^2^=2.221，*P*=0.329 > 0.05）。

**4:** 分组疗效对比 Efficacy comparison of the three groups

Group	*n*	Short-term efficacy							Long-term efficacy	
		CR	PR	SD	PD	ORR	DCR		Median of PFS	95%CI
1	21	2	13	4	2	71.43%	90.48%		12.4	9.9-14.9
2	16	1	9	5	1	62.50%	93.75%		12.7	12.2-13.2
3	5	1	2	1	1	60.00%	80.00%		15.8	7.9-32.5
CR: complete response; PR: partial response; SD: stable disease; PD: progressive disease; ORR: objective response rate; DCR: disease control response.										

**1:**
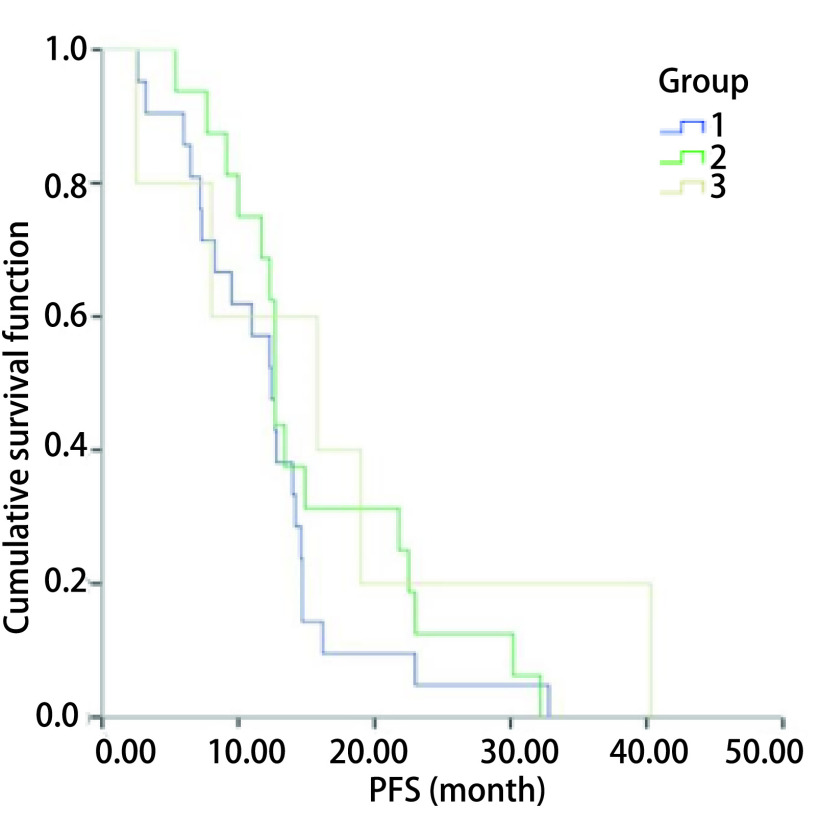
*Kaplan-Meier*法分析接受一代EGFR-TKI药物治疗的患者无进展生存期。组1为ddPCR及Super-ARMS检测*EGFR*基因突变结果均为阳性。组2为ddPCR及SUPER-ARMS检测EGFR基因突变结果均为阴性。组3为ddPCR检测结果为阳性而SUPER-ARMS检测结果为阴性 *Kaplan-Meier* analysis of progression-free survival in patients treated with first-generation EGFR-TKI drugs. Group 1 was the patient with positive results of ddPCR and super-arms testing for *EGFR* gene mutation; group 2 was the patient with negative results of ddPCR and super-arms testing for *EGFR* gene mutation; group 3 was the patient with positive results of ddPCR testing and negative results of super-arms testing. EGFR-TKIs: epidermal growth factor receptor-tyrosine kinase inhibitor

此外，有2例组织突变阴性但血浆突变阳性者分别接受了吉非替尼和埃克替尼治疗后，PFS分别为16.3个月及24个月。

## 3 讨论

随着基因技术的发展，靶向药物在晚期肺腺癌患者的治疗中起着越发重要的作用。然而因受患者心肺功能差无法取材、取材组织少或为坏死组织、病变位置特殊或质地坚韧活检钳不易着位等因素^[5]^的影响，从肿瘤组织处组织取材多有不易。部分患者因肿瘤组织取样艰难无法行靶向基因检测而错过靶向药物治疗机会。为解决这一问题，让更多患者从靶向药物治疗中获益，研究者们开始应用液体基因检测方法来代替组织基因检测^[6]^，哪一种液体基因检测方法更高效准确则逐渐成为了研究的热点。在2015年发布的《NSCLC血液*EGFR*基因突变检测中国专家共识》中，首次将ARMS技术作为一种成熟的液体检测技术进行推荐和推广。既往众多研究人员^[7-9]^将Super-ARMS技术与肿瘤组织基因检测技术进行对比，结果均显示Super-ARMS特异度较高，在98%-100%之间，但是其灵敏度较低，可能导致部分患者因检测结果呈现假阴性而无法接受靶向药物治疗。为解决此问题，本研究选择ddPCR技术进行研究对比，明确ddPCR技术在血浆检测*EGFR*基因突变方面的临床价值。

ddPCR技术首次被应用于肺癌*EGFR*突变基因检测是2015年^[9]^，曾有研究结果显示ddPCR技术在检测血浆游离肿瘤ctDNA时敏感度可达0.01%，明显高于Super-ARMS技术的0.2%^[10, 11]^。马丽等^[12]^的临床研究结果也表明ddPCR技术在灵敏度方面明显优于ARMS技术。在本研究中，ddPCR检测结果与组织检测结果符合率为82.4%，高于Super-ARMS技术的71.4%;灵敏度为74.1%，明显优于Super-ARMS技术灵敏度58.6%;ddPCR技术特异度为90.2%，同样高于Super-ARMS技术的特异度83.6%。这与既往研究中的结果相符合，也提示了ddPCR技术在不同实验室里操作结果的稳定性。

*EGFR*基因的突变位点主要位于外显子18-21上^[13]^，其中19号外显子的缺失（19-Del）以及21号外显子的L858R突变（L858R）是靶向药物疗效较好的主要位点。因此，我们也对单个特异突变基因的检测结果进行比较中。19-Del突变的检测中ddPCR结果与组织的符合率达91.4%，ARMS结果与组织符合率为74.3%。在L858R突变基因的检测结果中，ddPCR结果与组织的符合率达81.4%，同样高于Super-ARMS技术的符合率78.5%。相较而言，ddPCR比ARMS技术结果更灵敏，再次证明了ddPCR技术的优越性。并且在两个主要突变位点的检测中对19-Del突变基因检测的准确率更高。

此外，ddPCR技术与Super-ARMS技术相比较而言还具有以下两点优势：①系统简单，成本较低，操作简便，一般实验室即可完成; ②结果可绝对定量定量分析，无需依赖扩增曲线及循环阈值（Ct），结果判读简单。

既往有研究表示*EGFR*基因突变结果对晚期肺腺癌患者的无进展生存期（progression-free survival, PFS）有极大的影响^[14]^，而*EGFR*基因突变阳性患者可从EGFR-TKIs药物治疗中明显获益^[15, 16]^。最近的研究显示^[12]^，*EGFR*基因突变阳性者接受一代EGFR-TKIs药物治疗后中位PFS可达12.1个月，而*EGFR*基因突变阴性患者的中位PFS仅2.4个月。本研究结果显示：肿瘤组织标本*EGFR*基因突变阳性患者接受一代EGFR-TKIs药物治疗的中位PFS为12.4个月，与既往研究结果相符。同时组1的中位PFS也为12.4个月（95%CI: 9.9-14.9）与总体相比，无统计学差异（*P* > 0.05），表明ddPCR技术检测结果与组织学检测具有一致性。同时，为评估近期疗效，本研究计算并比较了三组的ORR与DCR，结果发现虽然组3的值均低于组1和组2，但的组间的比较结果无统计学意义（*P* > 0.05），进一步证明了血浆ddPCR检测结果与组织检测结果的一致性。

此外，本研究中发现比较特殊的两位患者，其组织检测*EGFR*突变基因结果为阴性，而血浆检测结果为阳性，同样接受了TKI药物治疗，分别为吉非替尼和埃克替尼，PFS则分别为16.0个月和24.1个月，均高于中位PFS，显示同样从EGFR-TKI药物中获益。

血浆ctDNA来源于肿瘤细胞，为原发肿瘤以及转移形成的肿瘤细胞破裂脱落的DNA片段，进入外周血液循环系统。ctDNA通常片段化为170 bp左右，在手术摘除肿瘤或者化疗之后会快速清除。因抽提ctDNA的过程中来自白细胞的正常DNA污染导致血液中ctDNA不一定与原发灶有一致性，通常可以全血离心的方法去除所有的细胞，以降低DNA污染，然后分析余下的血浆。同时本研究在取血液标本及组织标本时保持在24 h之内，这保证了组织和血浆的检测结果一致性和可参考性。但本研究仍存在不足之处，如：组4的患者例数为0，无法比较此组患者与其它组患者的用药效果，少量随访数据的丢失导致PFS研究结果可能存在一定的偏倚等。

综上所述，随着技术的发展，外周血ctDNA *EGFR*基因突变检测已成为晚期肺腺癌患者*EGFR*基因突变检测的一种趋势，可作为靶向药物选择以及疗效预测的可靠依据。此种方法，取材方便，特异度及灵敏度均较高，一方面可减少患者接受组织取样的痛苦，从而提高患者的依从性，另一方面可使更多患者从EGFR-TKI药物获益。因各种检测方法优劣不等，临床所选方法不同可能对患者的治疗和预后产生极大的影响。本研究证实了ddPCR技术具有高敏感度和特异度，相较于SUPER-ARMS技术具有更高的符合度，有望成为检测现有*EGFR*基因突变位点、指导晚期肺腺癌治疗以及疗效评估的一种可靠方式。
